# Southern Medical Students and Southern Medical Schools

**Published:** 1860-03

**Authors:** 


SOUTHERN MEDICAL STUDENTS AND SOUTHERN MEDICAL SCHOOLS.—In the January number of the Review we gave a brief account of the secession of some of the Southern medical students from the schools of this city; but we do not intend now to return to the subject, convinced that time will place the whole matter in its proper light.

In connection, however, with the topic, we deem it necessary to refer to an article, by Dr. Bowling, in the February number of the Nashville Journal of Medicine and Surgery, denying, on behalf of his colleagues, any participation, in the way of sympathy, with the late stampede among the Southern students of this city. The notice which we gave of that occurrence was solely of a historical character; and for the facts which guided us on the occasion, we were indebted to various sources of information, all of them, as we supposed, entitled to confidence. Our data in regard to the University of Nashville were founded partly upon the statements of members of the class of the Jefferson Medical College playing a prominent part in the stampede, and partly upon the annexed telegraphic dispatch, dated Nashville, December 20, 1860, and addressed to a medical student in this city:—

"Yes; come one, come all.
W. K. BOWLING"

Dr. Bowling, in the article referred to, states that no dispatch, or letter, from him was read at the students' meeting in this city on the twentieth of December. This assertion, it appears, was true, for we have just learned that the above dispatch was not received until a short time after the adjournment of the meeting. Be this, however, as it may, it reached here some time before their departure, and was no doubt extensively circulated.

In the article of our journal, above referred to, we included the schools at New Orleans as among those that had opened their arms to the disaffected students. It is due, however, to the University of Louisiana to say, that she stood entirely aloof from any participation in the movement. We make this statement upon the assertion of our colleague, Dr. Richardson, the Professor of Anatomy in that institution, who declares that the whole faculty were opposed to the proceeding, and that no dispatch, or communication of any kind, was sent by them to this city either before, during, or after the stampede. The Shelby Medical College of Nashville, we are happy to say, makes a similar disclaimer in the February number of the Nashville Medical Record.

